# Structural basis of Cas9 DNA interrogation with a 5′ truncated sgRNA

**DOI:** 10.1093/nar/gkae1164

**Published:** 2024-12-09

**Authors:** Kaitlyn A Kiernan, Jieun Kwon, Bradley J Merrill, Miljan Simonović

**Affiliations:** Department of Biochemistry and Molecular Genetics, University of Illinois Chicago, 900 S Ashland Ave, Chicago, IL 60607, USA; Department of Biochemistry and Molecular Genetics, University of Illinois Chicago, 900 S Ashland Ave, Chicago, IL 60607, USA; Department of Biochemistry and Molecular Genetics, University of Illinois Chicago, 900 S Ashland Ave, Chicago, IL 60607, USA; Department of Biochemistry and Molecular Genetics, University of Illinois Chicago, 900 S Ashland Ave, Chicago, IL 60607, USA

## Abstract

The efficiency and accuracy of CRISPR-Cas9 targeting varies considerably across genomic targets and remains a persistent issue for using this system in cells. Studies have shown that the use of 5′ truncated single guide RNAs (sgRNAs) can reduce the rate of unwanted off-target recognition while still maintaining on-target specificity. However, it is not well-understood how reducing target complementarity enhances specificity or how truncation past 15 nucleotides (nts) prevents full Cas9 activation without compromising on-target binding. Here, we use biochemistry and cryogenic electron microscopy to investigate Cas9 structure and activity when bound to a 14-nt sgRNA. Our structures reveal that the shortened path of the displaced non-target strand (NTS) sterically occludes docking of the HNH L1 linker and prevents proper positioning of the nuclease domains. We show that cleavage inhibition can be alleviated by either artificially melting the protospacer adjacent motif (PAM)-distal duplex or providing a supercoiled substrate. Even though Cas9 forms a stable complex with its target, we find that plasmid cleavage is ∼1000-fold slower with a 14-nt sgRNA than with a full-length 20-nt sgRNA. Our results provide a structural basis for Cas9 target binding with 5′ truncated sgRNAs and underline the importance of PAM-distal NTS availability in promoting Cas9 activation.

## Graphical Abstract

**Graphical Abstract:**
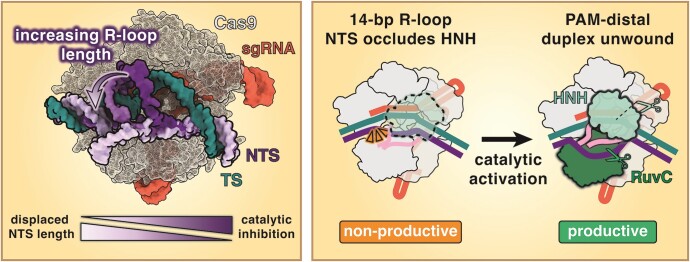

## Introduction

The Type II-A CRISPR-Cas9 system from *Streptococcus pyogenes* has been widely utilized for programmable genome manipulation (1–6). The effector nuclease, Cas9, is directed by a single guide RNA (sgRNA) that recognizes a 20 base-pair (bp) sequence that resides directly upstream of a short protospacer adjacent motif (PAM) site. Sufficient Watson–Crick base pairing of the sgRNA and the genomic target triggers conformational activation of Cas9 and results in the cleavage of both DNA strands. Reprogramming Cas9 is achieved by changing the complementary 20 nucleotide (nt) spacer at the 5′ end of the sgRNA (1). While this allows for easy manipulation of the system, it has been found that Cas9 can tolerate mismatches to the desired target site, especially in the 5′ region, which often leads to undesirable off-target editing (7–11). Given that Cas9 is currently being utilized in many therapeutic approaches to correct genetic mutations, it is crucial to ensure high fidelity of Cas9 targeting.

Many research groups have engineered Cas9 variants that reduce off-target cleavage, but often come at the expense of diminished on-target mutagenesis (12–15). An intriguing strategy for improving fidelity involves the modulation of sgRNA length and composition instead of engineering Cas9. Studies have shown that 5′ truncation of the sgRNA spacer to 17-nt or 18-nt results in enhanced discrimination between on- and off-target sites *in vivo* while maintaining comparable editing activity (16,17). As Cas9 can accommodate a wide range of mismatches in the PAM-distal heteroduplex, it has been speculated that removing the opportunity for those mismatches altogether may increase the penalty for mismatches in the shorter heteroduplex (16). On the other hand, truncation of the spacer past 15 nt inhibits catalytic activity *in vivo* while still maintaining on-target binding (18–20). When multiplexed with active sgRNAs, truncated sgRNAs enable simultaneous gene regulation, editing and reduction of off-target cleavage using a single active Cas9 (18–23). Although truncated sgRNAs are widely used for *in vivo* applications, it remains unclear how removing electrostatic interactions in the PAM-distal region enhances fidelity and why truncation past 15 nt prevents catalytic activation while still allowing persistent binding.

Previous structural studies of Cas9 containing consecutive mismatches in the PAM-distal region have provided mechanistic insight into our current model for Cas9 conformational activation (24,25). Accommodation of the PAM-distal RNA–DNA heteroduplex into the central channel of Cas9 allows for REC3 docking in positions +15–18 of the duplex and imparts a kink in the DNA. This DNA kinking is coupled to the undocking of the HNH domain from RuvC, enables the L1 linker to dock onto the heteroduplex, and facilitates the correct positioning of the nuclease domains. Notably, it has been shown that R-loop formation past 14–16 bp triggers undocking of the HNH nuclease domain and Cas9 adopts a catalytically competent conformation (25). While these structures contained shorter R-loops and were important for our understanding of R-loop propagation, they were determined using nuclease-dead Cas9 and retained the mismatched 5′ end of the sgRNA. Given that Cas9 was already inactivated, and it is unclear to what degree conformational activation is affected by having six consecutive mismatches in the sgRNA, these complexes may not represent the true inhibited conformation present during wild-type (WT) Cas9 DNA interrogation bound to fully truncated sgRNA.

To understand how truncation of the sgRNA prevents Cas9 cleavage, we carried out biochemical and structural characterization of Cas9 when bound to a truncated 14-nt sgRNA. Here, we use cryogenic electron microscopy (cryo-EM) to determine two structures of Cas9 in a pre-cleavage state and a nonproductive intermediate state. Our structures reveal that the unwound non-target strand (NTS) is primarily responsible for catalytic inhibition by physically occluding the HNH nuclease from docking onto the target strand (TS). We demonstrate that this inhibition can be alleviated by modulating DNA topology. Our findings provide a mechanistic understanding of how sgRNA truncation past 15 nt prevents catalysis and how PAM-distal DNA unwinding plays a critical role in DNA cleavage with truncated guides.

## Materials and methods

### mESC editing experiments

For Cas9 mutagenesis of 12 distinct genes in mouse embryonic stem cell (mESC), corresponding 20 or 14-nt sgRNA sequences were cloned into pSP-sgRNA (Addgene) (Supplementary Table S2). mESCs were transfected with a mixture of 175 ng pPGKpuro (Addgene), 175 ng pX330 (lacking sgRNA insert) and 175 ng of the relevant pSP-sgRNA plasmid. As a background control, a transfection containing an empty pSPgRNA was used alongside sgRNA-containing transfections. Two days after transfection, cells were split in the presence of 2 μg/ml puromycin, and selection was applied for 48 h. Genomic DNA was harvested 4 d after transfection and approximately 100 ng of DNA was used in polymerase chain reaction (PCR) to amplify respective target sites while attaching Illumina adaptor sequences for subsequent barcoding steps (Supplementary Table S3). PCR products were analyzed via agarose gel and submitted to the Genome Research Core (University of Illinois Chicago, Chicago, IL, USA) for Illumina MiniSeq analysis generating 150–300 bp paired-end reads. Sequencing data analysis for insertion/deletion (indel) frequency was carried out using CRISPResso2 (26) and additional information regarding sequences used is provided in supplementary data.

### Cas9 expression and purification

*Streptococcus pyogenes* Cas9 (SpCas9) constructs were cloned into a pET28b expression vector containing a C-terminal His_6_ tag. Recombinant Cas9 was expressed in *Escherichia coli* strain OverExpress C41(DE3) (Sigma) and purified by Ni-NTA (nickel-nitrilotriacetic acid) and ion exchange chromatography. Expression was induced when cells reached an OD_600_ of ∼0.6 by adding isopropyl β-D-1-thiogalactopyranoside to a final concentration of 0.2 mM. Cells were incubated for 18–20 h at +18°C while shaking and then pelleted at +4°C by centrifugation at 7000 r.p.m. for 30 min. The cell pellet was resuspended in lysis buffer [20 mM HEPES–NaOH, pH 7.5, 300 mM NaCl, 3 mM βME, 10% (v/v) glycerol] supplemented with a protease inhibitor tablet (Roche). Cells were sonicated on ice and the lysate was clarified at 15 000 r.p.m. for 45 min. Lysate supernatant was loaded onto a His-Trap FF crude column (Cytiva), washed with the lysis buffer containing 20 mM imidazole and eluted with the same buffer but supplemented with 350 mM imidazole. Subsequent purification was performed using a HiTrap SP Sepharose High Performance cation exchange column (Cytiva) and a linear gradient of 100–500 mM KCl in 20 mM HEPES–KOH, pH 7.5. Fractions containing Cas9 were pooled, concentrated to 10 mg/ml and buffer exchanged into Cas9 protein storage buffer [20 mM HEPES, pH 7.5, 200 mM NaCl, 1 mM EDTA, 10% (v/v) glycerol, 0.5 mM TCEP]. All purified samples were flash-frozen and stored at −80°C.

### Nucleic acid preparation

The 40- and 55-bp DNA substrates were prepared by annealing two complementary single-stranded DNA oligonucleotides (IDT; Supplementary Table S1). Each oligo was resuspended in water to a final concentration of 100 μM. For the 40-bp cryo-EM substrate, oligonucleotides were combined a 1:1 molar ratio and annealed by heating to +95°C for 5 min and slowly cooling to room temperature. For fluorescently labeled substrates, the unlabeled oligo was added in 1.2 molar excess during annealing. All sgRNAs used in this study were purchased from GenScript, resuspended in nuclease-free water to a final concentration of 20 μM, flash-frozen in liquid nitrogen and stored at −80°C. Sequences for oligonucleotides and the sgRNA scaffold are listed in Supplementary Table S1. For the plasmid cleavage assays, the same Cas9 target site that was used in the linear fragments was cloned into pUC19, prepared in bulk via the ZymoPURE II Plasmid Maxiprep kit, and concentrated with the Vacufuge Plus Vacuum Concentrator (Eppendorf).

### Thermal shift assay

Thermal unfolding profiles of Cas9 samples in 1× cleavage buffer at concentrations of 0.25 mg/ml were measured using the Tycho NT.6 (NanoTemper Technologies). Thermal unfolding profiles were generated by monitoring intrinsic fluorescence at 330 and 350 nm while heating the sample from +35 to +95°C. Initial fluorescence, the ratio of fluorescence (350/330 nm) and the Ti were calculated by Tycho NT.6. Data analysis was performed in GraphPad Prism version 10.3.1 (GraphPad Software, San Diego, California USA). Statistical significance between samples was determined using one-way ANOVA followed by the Dunnett multiple comparisons test.

### Microscale thermophoresis

Microscale thermophoresis (MST) reactions were performed in MST assay buffer [20 mM HEPES, pH 7.5, 200 mM NaCl, 10 mM MgCl_2_, 0.05% (v/v) Tween-20] at +37°C. The Cy5-labeled 40-bp double-stranded DNA (dsDNA) target was adjusted to 500 pM with MST assay buffer and dilution series were prepared according to the MO.Control software-protocol (NanoTemper Technologies). A series of two-fold dilutions of pre-formed Cas9 ribonucleoproteins (RNPs) were prepared in 10 μl of MST assay buffer and mixed with 10 μl of labeled target DNA. After 15 min, the samples were loaded into Monolith NT.115 Premium Capillaries and measured using the Monolith NT.Automated (NanoTemper Technologies) using 40% light-emitting diode (LED) excitation power and medium MST power.

### Cryo-EM sample preparation and data collection

Cas9 complex samples were prepared in complex buffer (20 mM HEPES, pH 7.5, 200 mM NaCl, 1.0 mM EDTA, 10 mM MgCl_2_, 0.5 mM TCEP) in a 1:1.5:1.5 molar ratio of Cas9:sgRNA:DNA. WT SpCas9 and 14-nt sgRNA were combined and incubated for 10 min at room temperature before adding the 40-bp dsDNA substrate. The ternary complex was incubated for 15 min at +37°C and filtered in a 0.22 μm spin filter. Samples were applied to UltrAuFoil 1.2/1.3 300 mesh holey gold grids (Quantifoil) plasma cleaned using H_2_/O_2_ gas mixture for 5 s in a Solarus plasma cleaner (Gatan) operating at 50 W. The sample was allowed to adsorb for 2 s before blotting for 6 s, followed by plunge freezing into liquid ethane cooled at liquid nitrogen temperature with a Leica EM GP (Leica Microsystems) and operating at +20°C and 90% humidity. Grids were sent to the National Cryo-Electron Microscopy Facility at the Frederick National Laboratory for screening and imaging. Imaging was carried out using a Titan Krios (FEI Company) operated at 300 kV and equipped with a GIF Quantum Energy Filter (Gatan) operating in zero-energy-loss mode with a slit width of 20 eV. Images were recorded with a K3 camera (Gatan) in super-resolution counting mode, with a defocus range between –1.0 to –2.5 μm at a nominal magnification of ×81 000 corresponding to a super-resolution pixel size of 0.56 Å. Automated data acquisition was carried out in Latitude software (Gatan) using 40-frame movies with intermediate frames recorded every 0.1 s. The dose rate of 16.66 e^−^/s/phys. pixel with a total exposure time of 3.8 s resulted in a nominal dose of ∼50 e^−^/Å^2^ per micrograph.

### Cryo-EM data processing

All data were processed in cryoSPARC v4.0 (27) (Supplementary Figure S3). Imported movies were subject to patch motion correction and patch contrast transfer function (CTF) estimation. Particles were picked automatically with blob picker and subject to multiple rounds of 2D classification to filter out junk particles. A subset of particles was used to seed a multi-class *ab initio* reconstruction for further sorting. All particles from 2D classification were then classified using heterogenous refinement with the *ab initio* volumes. The best resulting class was subject to multiple rounds of 3D classification using the principal components analysis (PCA) initialization mode with forced hard classification. One final round of nonuniform refinement (28) was conducted using per-particle defocus and global CTF optimization parameters to generate the final 3D reconstructions. Models 6O0Z and 7S4V were rigid body fit into State I and State II maps, respectively.

### Structural modeling

3D models were manually built and inspected in Coot (29) and ISOLDE (30), and structures were real-space refined in Phenix (31). All figures were generated in ChimeraX v1.6.1 (32). To generate AlphaFold 3 (AF3) models shown in Supplementary Figure S2, the sequences of SpCas9, sgRNAs and the corresponding target and nontarget DNA strands were uploaded to the AlphaFold server (33).

### Cas9 cleavage assays

All cleavage reactions were assembled in 1× cleavage buffer (20 mM HEPES, pH 7.5, 100 mM KCl, 0.5 mM TCEP, 10 mM MgCl_2_, 5% glycerol). To form the Cas9 RNP, recombinant Cas9 was mixed with annealed sgRNA in a 1:1.5 ratio and incubated at room temperature for 15 min. DNA was added to initiate the reaction at a molar ratio of 1:5 DNA:RNP. At each timepoint, an aliquot of the reaction was quenched by addition of 500 mM EDTA and 20 μg Proteinase K (Thermo Fisher Scientific). Plasmid cleavage products were resolved on a 1% (w/v) agarose gel in 1× TAE (40 mM Tris-acetate, 1 mM EDTA) buffer ran at 90 V for 75 min and post-stained with 3X GelRed (Millipore Sigma). Agarose gels were imaged using the Azure 400 (Azure Biosystems). Quantification of linear product was determined by densitometry analysis in ImageJ (34). For the plasmid cleavage assays used to determine catalytic rate, total linear product was normalized to EcoRI-digested plasmid DNA. Cas9 cleavage products using fluorescently labeled 55-bp linear fragments were resolved on a 15% (w/v) TBE-Urea (7 M) polyacrylamide gel and ran at 180 V for 45 min. TBE-Urea gels were imaged with the ChemiDoc MP (Bio-Rad). Uncropped gel images are provided in Supplementary Figures S6 and S7.

### Kinetic analysis

The catalytic rate for plasmid cleavage assays was determined using GraphPad Prism Version 10.3.1 (GraphPad Software, San Diego, California, USA).

For Cas9 reactions with a full-length 20-nt sgRNA, product formation over time was fit using the double exponential equation:


\begin{equation*}Y = A*(1-{{\mathrm{e}}^{ - k1t}}) + {k_2}{\mathrm{t}}\end{equation*}

where *Y* represents the concentration of product, *A* represents the amplitude, *k_1_* represents the observed catalysis rate *k*_fast_ for the initial burst phase of the reaction and *k_2_* represents the observed catalysis rate *k*_slow_ for the linear phase of the reaction.

For reactions with 14-nt sgRNAs, product formation over time was fit using a single exponential equation:


\begin{equation*}Y = A*\left( {1-{{\mathrm{e}}^{ - kt}}} \right)\end{equation*}

where *Y* represents the concentration of product, *A* represents the amplitude and *k* represents the observed catalysis rate *k*_obs_.

## Results

### DNA editing with truncated sgRNAs in mESCs

**Figure 1.**
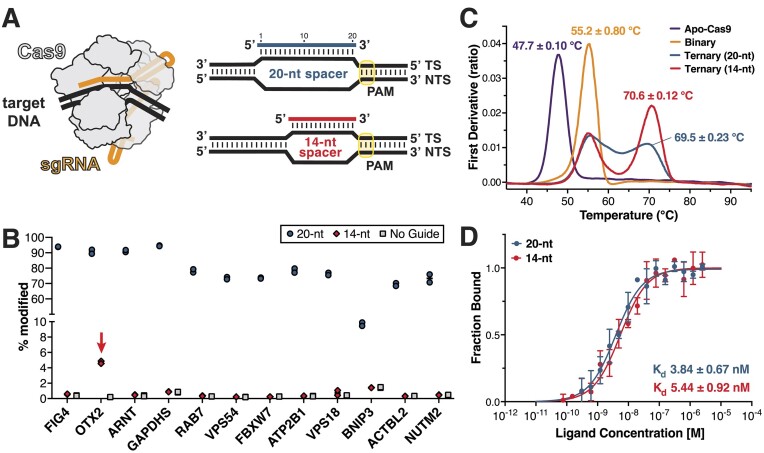
Biochemical characterization of Cas9 bound to a 14-nt sgRNA. (**A**) Cartoon of the Cas9 ternary complex and schematics of the R-loop consisting of the DNA TS, NTS and a 20- or 14-nt spacer sequence. (**B**) Indel generation using a 20-nt sgRNA, 14-nt sgRNA or a no guide control measured with targeted amplicon next-generation sequencing. Percent (%) modified reads were quantified with CRISPResso2 (26). Data are represented as the mean ± S.D. across two biological replicates. Editing with a 14-nt sgRNA above background level is indicated with an arrow. (**C**) Thermal unfolding profiles of Cas9 alone (apo), bound to sgRNA alone (binary) or bound to a 40-bp DNA substrate with either a 20-nt spacer or 14-nt spacer (ternary). Values are plotted as the first derivative of the ratio of fluorescence at 350/330 nm. Measurements are performed in triplicate and shown as mean ± S.D. (**D**) Binding curves of Cas9 RNPs with either a 20-nt spacer or 14-nt spacer determined using MST. Data are represented as the mean ± S.D. from three independent replicates and fit using the *K*_d_ model in the MO.AffinityAnalysis software.

While previous studies have shown a sharp decrease in Cas9 editing activity using sgRNAs shorter than 16-nt, this has yet to be systematically benchmarked across a wide range of genomic targets and is particularly pertinent since variability in Cas9 editing efficiency is often highly dependent on sequence context. We therefore analyzed the editing capability of Cas9 programmed with either 20- or 14-nt sgRNAs at 12 different previously characterized loci in mESCs (Supplementary Tables S2 and S3) (35). Whereas 20-nt sgRNAs enabled high editing efficiency (>70%) across all loci (Figure 1B), the truncated 14-nt sgRNAs were generally insufficient for generating indels at the majority of selected targets. Surprisingly, we observed that OTX2 was edited with ∼5% editing frequency, which was higher than the background level observed in the non-targeting control (Figure 1B; Supplementary Figure S5). This observation critically illustrates that truncated sgRNAs, even when $ \le$16 nt in length, can still mediate gene editing in mammalian cells and necessitates further characterization given that these guides are being implemented as catalytically inactive complexes.

### Truncation of the sgRNA to 14 nt does not impact complex stability or affinity

Since we established that the truncated 14-nt guide was able to mediate, albeit inefficiently, DNA editing, we investigated the impact of guide truncation on Cas9 gRNA stability and targeting efficiency. We first measured thermal unfolding profiles of apo-Cas9, a Cas9:sgRNA binary complex, and a ternary Cas9:sgRNA:dsDNA complex with either a 20-nt spacer or 14-nt spacer with the aim to investigate the impact of the truncated guide on the overall gRNP complex stability (Figure 1A). While apo-Cas9 exhibits relatively low thermal stability with an inflection temperature (*T*_i_) of +48°C, addition of sgRNA increases the *T*_i_ to ∼+55°C (Figure 1C). The addition of a complementary dsDNA substrate causes a second shift in *T*_i_, further enhancing complex stability. Ternary complexes bound to guides with 20- and 14-nt spacers show comparable stability with *T*_i_ values of ∼+69°C and ∼+71°C, respectively (Figure 1C). The lack of decrease in *T*_i_ values indicates that even with truncation of the sgRNA, overall complex integrity is sufficiently maintained.

We then used MST to determine the relative binding affinities of Cas9 to its DNA target when bound to either a full-length 20-nt or truncated 14-nt spacer. Our measurements show that Cas9 affinity for its complementary target is 3.84 nM when bound to a 20-nt sgRNA and 5.44 nM when bound to a 14-nt sgRNA (Figure 1D). At this low-nM range, a difference of 1–2 nM in binding affinity is not likely to impact the DNA targeting ability of Cas9 and that the defect in DNA editing is likely caused by other factors.

### Structural characterization of Cas9 bound to a 14-nt sgRNA

Having seen no meaningful difference in complex stability or binding affinity between the 20- and 14-nt bound complex and given the success of CRISPRi with 14-nt sgRNAs, we hypothesized that guide truncation must prevent conformational activation of Cas9 after binding the DNA target. To capture the inhibited state, we employed cryo-EM to determine the structure of Cas9 bound to a 14-nt sgRNA. We chose to use the same λ1 target DNA sequence that was used in previous studies, thus enabling direct comparison of our structures with previously characterized Cas9 complexes (24,25,36–39). WT Cas9 was reconstituted with an sgRNA containing a 14-nt spacer upstream of the 5′ NGG 3′ PAM sequence (Figure 1A). The pre-formed Cas9 gRNP was added to a 40-bp dsDNA target in catalytically competent buffer conditions containing 10 mM Mg^2+^. To ensure complete complex formation, samples were allowed to equilibrate at +37°C for 15 min before quenching the reaction via vitrification.

**Figure 2.**
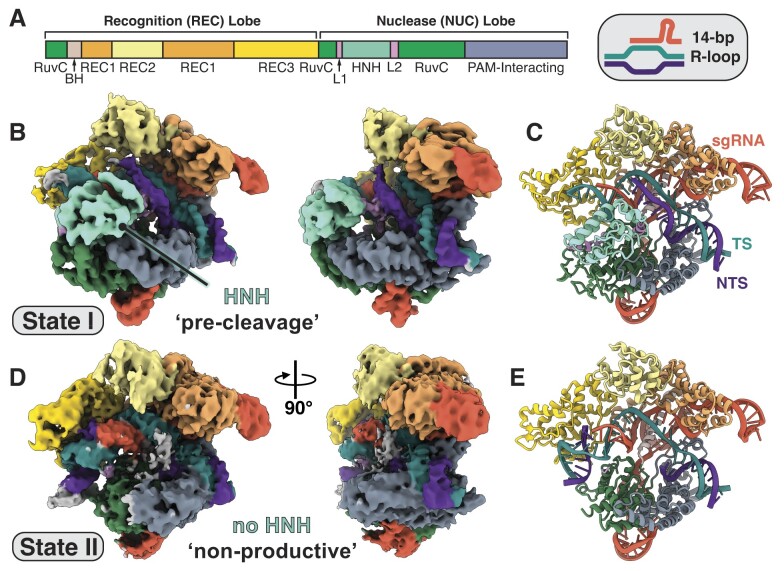
Structures of the Cas9 ternary complex. (**A**) Domain organization of Cas9 that consists of the recognition (REC) and nuclease (NUC) lobes. The same color coding is used in representation of 3D maps and structures shown in panels (B)–(E). (**B**) Unsharpened cryo-EM map of state I where the HNH nuclease domain is in a pre-cleavage conformation. (**C**) A ribbon diagram of the structure of the pre-cleavage state before nuclease lobe reorganization. (**D**)> Unsharpened cryo-EM map of state II. Loss of HNH density indicates it has undocked from the complex but exists in a nonproductive conformation. (**E**) A ribbon diagram of the structure of the nonproductive conformational state.

**Figure 3.**
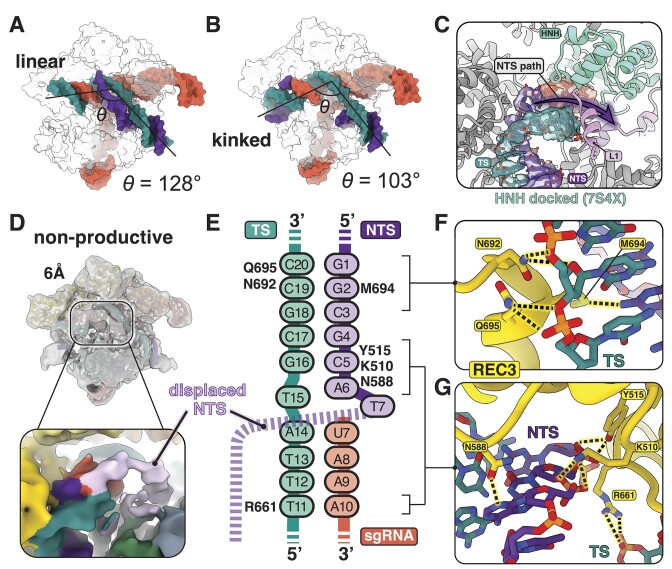
The displaced NTS path prevents Cas9 activation. (**A**–**B**) Comparison of the RNA–DNA heteroduplex conformation where the angle (*θ*) represents the angle of the PAM-distal duplex relative to the PAM-proximal duplex. (A) The heteroduplex is in a linear conformation in the pre-cleavage state. (B) Full accommodation of the duplex into the central channel imparts a kinked conformation of the duplex in the nonproductive state. (**C**) A zoom-in view of the PAM-distal R-loop in the nonproductive state. The HNH domain from an active Cas9 structure (PDB 7S4X) is overlayed with the nonproductive state and shows how the path of the unmodeled NTS (purple arrow) would directly block the HNH L1 linker (pink) from docking on the heteroduplex. (**D**) Low-pass filtered map of the nonproductive state at 6-Å resolution reveals density for the displaced NTS. A close-up view of its path is shown in the box below. (**E**) A schematic diagram of the PAM-distal R-loop showing REC3 interactions with both TS (cyan) and NTS (purple). For clarity, sgRNA numbering begins at position 7 where it has been truncated in our sample. (**F**) Close-up view of interactions between REC3, the sugar-phosphate backbone of the TS and the second nucleotide of the NTS. (**G**) Close-up view of interactions of REC3 with the DNA duplex directly upstream of the sgRNA truncation.

Cryo-EM data analysis revealed the existence of two distinct conformational states that were refined to global resolutions of 3.0Å and 3.1Å, respectively (Figure 2B and D; Supplementary Figures S3 and S4 and Supplementary Table S4). Representing ∼50% of the particles used for the final reconstructions, state I resembles an inactive state where the nuclease lobe has not undergone reorganization to adopt a catalytically competent state and is consistent with previously determined inactive structures (Supplementary Figure S1) (24,25,39). The HNH domain remains docked onto RuvC (Figure 2B) and we find that the sgRNA:DNA heteroduplex exhibits a linear conformation situated between the REC3 and RuvC domains (Figure 3A). A linear duplex conformation represents a state preceding REC3 docking onto the PAM-distal duplex and subsequent DNA kinking, which is required for conformational activation of the enzyme (24,25,39). Accordingly, we refer to state I as the ‘pre-cleavage’ state.

Notably, in this pre-cleavage state, we can visualize most of the unwound NTS. As with other structures determined during R-loop formation, the path of the NTS in our structure confirms that it runs parallel to the heteroduplex before being accommodated into the RuvC active site (Supplementary Figure S1) (25). We observed electrostatic interactions between residues Q771 and K775 of the L1 linker and G_5_ and A_4_ of the PAM-proximal NTS (Supplementary Figure S1) and suggest that the PAM-proximal NTS facilitates the stabilization of the α-helical L1 linker before nuclease lobe reorganization in this complex. Additionally, we observe density between a surface-exposed helix of the REC2 domain and the NTS backbone which indicates that REC2 may play an initial role in stabilizing the NTS during R-loop formation (Supplementary Figure S1). Our demonstration that the REC2 domain stabilizes the NTS in an initial conformation parallel to the heteroduplex corroborates previous single-molecule Förster resonance energy transfer (FRET) data that proposed the NTS exists in two discrete conformations, one in close proximity to the REC2 domain before and one that has been displaced farther into the central channel (40).

### The PAM-distal dsDNA duplex is accommodated through the central channel

Given the inefficiency of DNA editing with 14-nt guide, we anticipated that the Cas9 complex with a truncated guide would primarily adopt the pre-cleavage conformation. To our surprise, the remaining ∼50% of particles exhibit a markedly different conformation where the PAM-distal dsDNA is accommodated into the main binding channel and HNH becomes disordered. Because the DNA is intact and we do not observe either nuclease domain in a productive conformation, we refer to this structure as the ‘nonproductive’ state. In the nonproductive state, the PAM-distal duplex wedges deeper into the positively charged central channel formed by the REC3 and RuvC domains. When compared with the linear heteroduplex observed in the pre-cleavage state, the DNA target bends by an angle of 25° and adopts a kinked conformation, and the TS shifts ∼22 Å downward, closer to the NUC lobe (Figure 3B). DNA bending in the PAM-distal region is coupled to the structural disorder of the adjacent L1 and L2 linkers, which presumably triggers undocking of the HNH domain that is required for proper positioning of HNH and RuvC onto the TS and NTS, respectively (24,25,39). Interestingly, we find diffuse density for HNH and both linkers that is indicative of molecular motion in this region. It has been established that nuclease activation involves large coordinated domain movements and that the HNH domain exhibits a high degree of conformational flexibility during catalysis (39,24,25,38,37,41–47). The observed flexibility of the NUC lobe in the nonproductive state suggests that even with a shorter R-loop, accommodation of the PAM-distal DNA and subsequent kinking is sufficient to undock HNH.

Accommodation of the PAM-distal DNA duplex is facilitated by REC3 docking via several electrostatic interactions (Figure 3E–G). When compared with other Cas9 structures where REC3 has docked onto RNA–DNA duplexes longer than 14 bp (39,24,25,42,48), we observe the typical interactions where R661, N692 and Q695 make contact with the TS sugar-phosphate backbone. However, instead of establishing the usual contacts with the 5′ end of the sgRNA spacer, REC3 now interacts with the NTS in its place (Figure 3E). Side chains of K510 and Y515 are within H-bonding distance from the NTS sugar-phosphate backbone, whereas side chains of N588 and M694 establish H-bonds with the nucleobases A6 and G2, respectively (Figure 3F and G). Because the NTS remains hybridized in regions +16–20, the overall shape of the DNA duplex mimics that of the typical full-length RNA–DNA heteroduplex and allows for establishment of the same REC3 contacts observed in a 20-bp R-loop. Retention of these contacts promotes DNA kinking and allows for HNH undocking as observed in other catalytically active structures.

### The displaced NTS of the 14-bp R-loop prevents conformational activation of Cas9

Given that many of the pre-requisites for Cas9 conformational activation had been met (R-loop formation, DNA kinking and HNH undocking), it was clear to us that some other feature of this complex was responsible for catalytic inhibition. In other Cas9 structures containing short R-loops (25,49), the density for the PAM-distal R-loop is largely ambiguous due to flexibility of the duplex in that region. In our structures, however, we observe clear density for the PAM-distal region, including much of the displaced NTS that is normally too flexible to be captured in cryo-EM reconstructions (Figure 3D).

In the nonproductive state, we observe a distortion of the DNA duplex near positions +15 and +16 of the R-loop, the segment where the NTS rehybridizes with the TS (Figure 3E). Rehybridization of the NTS in this position results in an altered path for the unwound NTS, which is now routed over the heteroduplex in positions +12–14. Importantly, previous structures of catalytically active Cas9 complexes have shown that the L1 linker helix binds the minor groove of the heteroduplex in this same 12–14-bp region (39,24,25,42). We find that this minor groove of the heteroduplex is occluded by the new NTS route and would directly interfere with L1 linker interactions (Figure 3C).

We did not detect a conformation resembling the active state in our dataset, and as evidenced by a high degree of disorder in the nuclease lobe and NTS-mediated inhibition of L1 docking, the HNH and RuvC domains cannot adopt active conformations in this complex. Taken together, our data suggest that the occlusion of HNH docking causes inefficient conformational rearrangement of the nuclease domains. This then prevents proper positioning of the TS and NTS into the HNH and RuvC active sites and ultimately inhibits catalysis.

### R-loop formation past 14 bp alters the displaced NTS route

As the NTS is often not resolved in Cas9 structures, we used AF3 to compare the NTS path across R-loops of different lengths (Supplementary Figure S2) (33). In the AF3-predicted model of the 14-bp R-loop, the displaced NTS takes the same route as in our nonproductive state structure. Interestingly, the addition of one more base pair completely changes the predicted path of the NTS (Supplementary Figure S2). In the predicted 15-bp R-loop model, the NTS runs deeper into the central channel where it threads through the RuvC domain and out of the way of the L1 docking site. The same NTS path observed in the 15-bp complex is also observed in AF3-predicted complexes containing 14 complementary base pairs but consecutive mismatches in positions +15–20 of the duplex (i.e. the bubbled substrate used in our study that permits cleavage) (Supplementary Figure S2).

Closer examination of the R-loop in position +15 revealed that this region is exactly where REC3 begins to contact the PAM-distal duplex. In a 14-bp R-loop, there is enough space between the 5′ end of the guide and REC3 to accommodate the NTS in position +15, allowing the dsDNA duplex to remain hybridized in positions +16–20. However, when the guide is extended to 15 nt, electrostatic interactions of REC3 residues (K510, Y515 and N588) with the sgRNA sugar phosphate backbone in position +15 does not leave enough space for the NTS to take the same path as it does in the 14-nt complex (Supplementary Figure S2). Thus, we propose that, in addition to its established role in DNA bending and mismatch sensing, REC3 also plays a role in controlling the path of the displaced NTS in R-loops longer than 14 bp.

### DNA topology governs Cas9 activity with truncated sgRNAs

**Figure 4.**
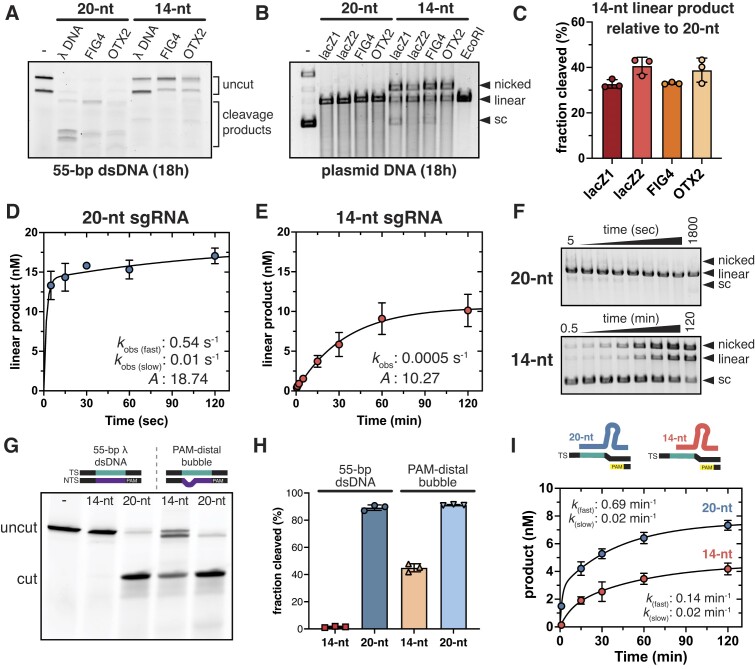
Cas9 cleavage of target DNA mediated by a 14-nt sgRNA. (**A**) Cas9 cleavage of linear 55-bp dsDNA targets when programmed with 20- or 14-nt sgRNAs. (**B**) Plasmid cleavage by Cas9 programmed with 20- or 14-nt sgRNAs. Cleavage was tested across four different target sequences and normalized to fully linearized plasmid by EcoRI. (**C**) Quantification of plasmid cleavage shown in panel (B). Data shown as the fraction of linear product generated by the 14-nt sgRNA complexes relative to the 20-nt sgRNA samples. (**D** and **E**) Observed plasmid cleavage rates (*k*_obs_) for (D) 20-nt sgRNA and (E) 14-nt sgRNA samples. (D) In the 20-nt samples, linear product was plotted over time (5–1800 s) and catalytic rates were determined using a double exponential equation. Reported values represent the burst (fast) and linear (slow) phase reaction rates and the amplitude (A) of the curve. (E) Linear product for the 14-nt samples was plotted over time (0.5–120 min) and the catalytic rate was determined using a single exponential equation. (**F**) Representative gels for the plasmid cleavage kinetics shown in panels (D)–(E). (**G**) Representative gel showing Cas9 cleavage after 2 h with a fully complementary dsDNA target or a bubbled dsDNA target containing consecutive mismatches from positions 15 to 20 in the PAM-distal duplex. (**H)** Quantification of DNA cleavage observed in panel (G). (**I**) PAMmer cleavage rates with either a 20- or 14-nt sgRNA. Rates determined as described for panels (D)–(E). (A–I) Data in all cleavage experiments are represented as the mean ± S.D. from three independent replicates. All cleavage products were quantified by densitometry analysis in ImageJ (34).

Our structures indicate that a shorter displaced NTS changes the path in the R-loop and prevents catalytic activation. Torsional stress generated from supercoiling can induce local melting of the dsDNA (50,51) and increase accessibility for nucleic acid binding proteins (52–55). Accordingly, we hypothesized that changing substrate topology would both increase the likelihood of PAM-distal unwinding and alleviate the inhibition. We tested the ability of a 14-nt sgRNA to activate Cas9 cleavage with a torsionally unrestrained linear substrate or a supercoiled plasmid substrate. When a 55-bp linear fragment was used, we did not observe DNA cleavage with 14-nt sgRNAs with any tested target, including the target that was edited in cells (Figure 4B; Supplementary Table S1). However, when a supercoiled substrate was utilized, Cas9 was able to cleave plasmid DNA with 14-nt sgRNAs across all targets tested, albeit at a much slower rate (Figure 4A). When compared with the cleavage rate with a 20-nt sgRNA (0.54 per s), DNA cleavage with a 14-nt sgRNA (0.0005 per s) was ∼1000-fold slower. This dramatic difference in cleavage rate can explain the disparity observed in Cas9 gene editing efficiency when programmed with a 14-nt sgRNA.

Because we observed that the path of the PAM-distal NTS is altered in the 14-nt complex, we tested whether DNA cleavage of a linear substrate would be rescued by artificially unwinding the PAM-distal end of the dsDNA target. To this end, we tested Cas9 cleavage of a fully matched 55-bp dsDNA substrate versus a bubbled substrate containing mismatches from positions 15–20 (Supplementary Table S1). Consistent with our previous findings, the 14-nt sgRNA was not sufficient for mediating cleavage of the fully matched linear substate. In contrast, we do find that this complex can cleave the bubbled substrate and confirms that PAM-distal DNA unwinding is sufficient for promoting catalytic activation of Cas9, even in the presence of a shorter R-loop (Figure 4G and H).

To further investigate the contribution of PAM-distal DNA unwinding in cleavage efficiency, we tested cleavage of a PAM-presenting oligonucleotide (PAMmer) containing a partially duplexed substrate with the PAM recognition sequence where the target region is single-stranded upstream of the PAM. In this setup, the DNA unwinding rate is negligible and differences in cleavage rates can be attributed to changes in conformational reorganization. We found that when compared with the ∼1000-fold reduction in cleavage rate on plasmid DNA, the 14-nt sgRNA complex only exhibited five-fold slower cleavage than a 20-nt bound complex (Figure 4I). This supports a model where in a 14-bp R-loop, the displaced NTS is primarily responsible for catalytic inactivation of Cas9 by physically occluding the path for HNH positioning on the TS scissile phosphate.

## Discussion

**Figure 5.**
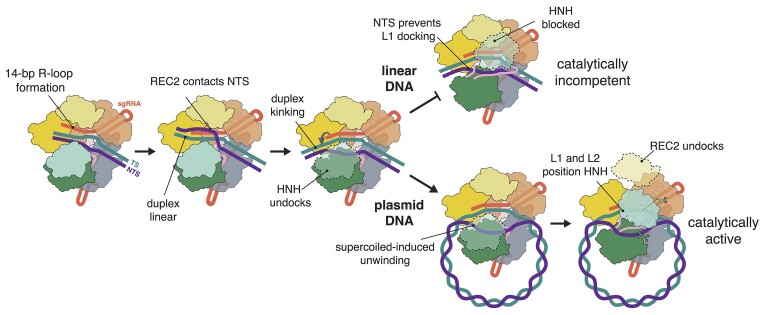
Proposed model of DNA cleavage with a 14-nt sgRNA. Upon formation of the 14-bp R-loop, the displaced NTS runs parallel to the linear sgRNA-TS heteroduplex and contacts the REC2 domain. As the PAM-distal dsDNA duplex is accommodated in the central channel between the REC3 and RuvC domains, the duplex adopts a kinked conformation. The kinking of the PAM-distal DNA facilitates undocking of the HNH domain. When the DNA substrate is linear, the NTS remains hybridized to the TS in the PAM-distal duplex, thus blocking the L1 linker from docking onto the minor groove of the heteroduplex. This, in turn, prevents both the correct positioning of the HNH domain and a complete nuclease lobe reorganization, ultimately inhibiting catalytic activation. When the DNA substrate is a plasmid, supercoiling-induced unwinding of the PAM-distal duplex alters the path of the NTS, allowing its accommodation into the cleft between the HNH and RuvC domains. This causes restructuring of the L1 and L2 linkers and permits docking onto the heteroduplex. Linker docking precisely positions the HNH and RuvC domains onto the TS and NTS, respectively, and enables full catalytic activation of Cas9.

Truncated sgRNAs have emerged as a powerful tool to simultaneously mediate on-target and block off-target Cas9 editing (19–23). They operate under the premise that Cas9 does not induce double-strand breaks (DSBs) when programmed with guides containing spacers shorter than 16-nt (18,19). Being primarily characterized *in vivo*, it has not been clear how truncation of the guide affects DNA cleavage efficiency at a mechanistic level. Our study begins to fill this significant gap in knowledge on how 5′ truncation of the sgRNA impacts catalytic activation of Cas9 (Figure 5).

Remarkably, we find that Cas9 is capable of editing in mESCs with 14-nt sgRNAs. Reduced catalytic activity contributes to observed poor editing efficiency, but it is critical to note that this effect may be context-specific across targets and could be higher than expected for some loci. Through biochemical and structural characterization, we show that Cas9 catalysis is inhibited when bound to a 14-nt sgRNA by blocking the reorganization of the NUC lobe. A shortened R-loop changes the trajectory of the NTS and physically prevents docking of the L1 linker onto the TS-sgRNA heteroduplex. Supercoiling-induced opening of the PAM-distal duplex unwinds the NTS enough to enable docking of the L1 and L2 linkers, proper positioning of the HNH and RuvC domains, and eventually DNA cleavage. This slow isomerization of the NUC lobe leads to extremely slow cleavage rates and explains why truncated sgRNAs are particularly inefficient at mediating DNA cleavage *in vivo*.

Biophysical studies investigating Cas9 conformational dynamics during target recognition and cleavage have established that catalytic activation proceeds via multiple discrete intermediate states, with the cleavage-competent state being a transiently sampled high-energy state (45,47,56). All-atom molecular dynamics (MD) experiments with Cas9 complexes have shown that HNH conformational activation is correlated with accommodation of the NTS into the RuvC domain (57). MD and smFRET experiments have also proposed that the NTS exists in two main conformations corresponding to active and inactive conformations, and that PAM-proximal NTS interactions with the L2 linker could promote catalytic activation of the enzyme (43,45,57). Our structural findings that the NTS physically blocks docking of the HNH domain in short R-loops provides direct evidence supporting these MD simulations and smFRET observations. Our new high-resolutions structures could thus be used to further improve the accuracy of predictive models of Cas9 activation.

In addition, it has been shown that mismatches to the target, especially in the PAM-distal region, affect the population distribution of Cas9 intermediate states and make sampling of the active state less likely (41,45,47). Within this context, we speculate that our nonproductive state structure may represent an inactive low energy state where the majority of the particles are trapped in a cleavage-incompetent conformation due to the altered path of the NTS. Our kinetics data further corroborate this model given that we do not observe full cleavage of either plasmid or PAMmer substrate, even with 18 h of incubation and five-fold excess active enzyme fraction. This phenomenon has also been observed where Cas9 programmed with mismatched spacers accumulate nicked intermediates and is correlated with the length of consecutive mismatches in the 5′ end of the sgRNA (58).

It is worth noting that many of the high-fidelity Cas9 variants contain mutations in the same residues we observed making contact with the PAM-distal NTS in our nonproductive structure (12,14,59,60). These REC3 residues are critical for sensing distortions in the PAM-distal R-loop and have been shown to significantly reduce cleavage rate of mismatched targets independent of DNA unwinding rate (15). In addition, structures of Cas9 in complex with consecutive mismatches in positions +15–17 show that conformational activation of Cas9 is prevented due to inefficient DNA kinking (24). These data support a model where distortion of this region of the R-loop would prevent DNA kinking by disrupting REC3-mediated contacts, thus rationalizing why mutations in this region of Cas9 yield low cleavage rates independent of DNA unwinding. Because the PAM-distal DNA duplex maintains contact with these mutation-sensing residues in our structure, and is observed in a kinked conformation, the mechanism of catalytic inhibition likely differs from that observed in the engineered high-fidelity variants. Our model instead suggests that when bound to a truncated sgRNA, the penalty for additional mismatches (i.e. further distortion of the R-loop) would be much higher and therefore even less likely to sample the active state. Mismatches to the 5′ end of a truncated sgRNA may favor NTS rehybridization in the PAM-distal duplex and would render truncated sgRNA complexes particularly vulnerable to NTS-mediated inhibition. Thus, the mechanism we describe for Cas9 inhibition with truncated guides provides a possible explanation for how 5′ truncation can enhance editing fidelity *in vivo*.

Local supercoiling density has been shown to impact Cas9 promiscuity in both binding and cleavage of the DNA target (47,61–63). Mechanical stretching and negative supercoiling can increase the tolerance for mismatches and presumably lowers the threshold for achieving the conformational checkpoint required for cleavage (63). These reports correlate well with our finding that catalytic activation of Cas9 programmed with a 14-nt sgRNA can only be achieved *in vitro* when a supercoiled plasmid or if the NTS is artificially unwound or removed. DNA sequence context also plays a large role in DNA bending and local DNA bubble formation (64). AT-rich regions in DNA duplexes are inherently more prone to melt and has been shown to influence Cas9 cleavage rate with a 16-bp R-loop (65). This propensity for local DNA melting can be further accentuated by increasing GC-content in adjacent regions (64). Intriguingly, the genomic target that was edited by the 14-nt sgRNA in our *in vivo* work has a high degree of PAM-distal GC content and presents an attractive avenue for further exploration.

Our observation that Cas9 can cut a supercoiled plasmid with a 14-nt guide *in vitro*, but the same efficiency was not observed for genomic DNA raises a question concerning the relative contribution of supercoiling versus sequence context in mediating DNA cleavage. One plausible explanation for this discrepancy is that the catalysis rate with the 14-nt sgRNA is significantly slower than that with full-length guides, and at most genomic loci, would not be fast enough to produce the DSBs necessary for indel formation. In this context, a limitation of our study is that we only assessed cleavage efficiency *in vitro* using a single spacer sequence. Future studies investigating different degrees of DNA supercoiling and sequence-dependent effects on Cas9 activity will be crucial to fully understand the mechanistic nuances contributing to cleavage efficiency with truncated guides.

Collectively, our study offers a molecular explanation for how 5′ sgRNA truncation impacts Cas9 cleavage. Our results underscore the importance of PAM-distal DNA unwinding and the influence of substrate topology in the conformational control of Cas9. These findings advance our understanding of the elements which precisely control Cas9 enzyme activity and will be critical to consider when designing safe applications of Cas9-based technologies.

## Supplementary Material

gkae1164_Supplemental_Files

## Data Availability

All sequencing data for this manuscript have been submitted to the SRA database with associated BioProject accession number PRJNA1127704. Atomic coordinates and cryo-EM maps have been deposited in the Protein Data Bank under accession numbers 9C9P (pre-cleavage state) and 9CGU (nonproductive) and in the Electron Microscopy Data Bank under accession codes EMD-45368 (pre-cleavage) and EMD-45586 (nonproductive). Source data are available.

## Notes

Present address: Miljan Simonović National Institute of General Medical Sciences, National Institutes of Health, Bethesda, MD 20892, USA.

Present address: Kaitlyn A. Kiernan University of Texas at Austin, Department of Molecular Biosciences Austin, TX 78712, USA.
