# Supplementary material for: Structural basis of Cas9 DNA interrogation with a 5′ truncated sgRNA
Source: Nucleic Acids Res. 2024 Dec 9;53(1):gkae1164. doi: 10.1093/nar/gkae1164 (PMC11724282; doi:10.1093/nar/gkae1164)
Supplement: gkae1164_Supplemental_Files [file gkae1164_supplemental_files.zip › Supplementary_Data_Revised.pdf]

Supplementary Data

Structural basis of Cas9 DNA interrogation with a 5' truncated sgRNA

Kaitlyn A. Kiernan[*,1], Jieun Kwon, Bradley J. Merrill, Miljan Simonović[*,#]

Department of Biochemistry and Molecular Genetics, University of Illinois Chicago, Chicago, IL 60616, USA

[#] Present address: National Institute of General Medical Sciences, National Institutes of Health, Bethesda, MD 20892, USA.

[1] Present address: University of Texas at Austin, Department of Molecular Biosciences Austin, TX 78712, USA.

* Corresponding authors:

Miljan Simonović, PhD
National Institutes of Health
National Institute of General Medical Sciences
Bethesda, MD 20892
USA
Email: miljan.simonovic@nih.gov

Kaitlyn Kiernan
University of Texas at Austin
Department of Molecular Biosciences
Austin, TX 78712
USA
Email: kaitlyn.kiernan@austin.utexas.edu

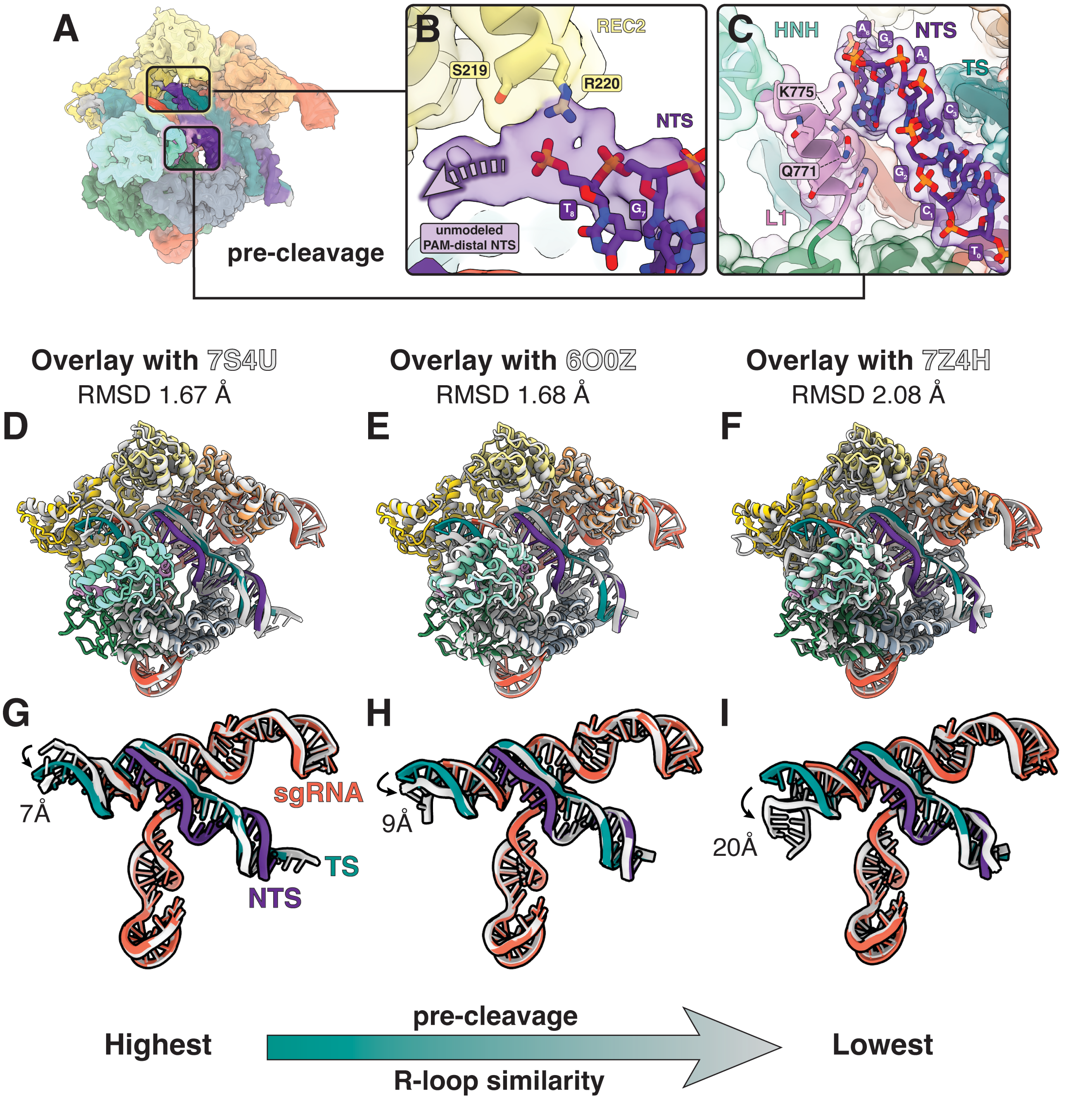

Supplementary Figure 1. Structural comparison of the pre-cleavage state. (A) The unsharpened cryo-EM map of the pre-cleavage state. (B) Zoom-in view of interactions between α-helix of the REC2 domain and the NTS backbone. (C) Zoom-in view of the interactions between the PAM-proximal NTS and L1 linker. (D-F) Superimposition of the pre-catalytic state structure with (D) Cas9 bound to a mismatched target in positions +12-14 (PDB 7S4U), (E) Cas9 in the pre-catalytic state (PDB 6O0Z), and (F) Cas9 bound to a 14-bp R-loop containing mismatches in positions +15-20 (PDB 7Z4H). (G-I) Cartoon representations of the R-loop structure in each of the superimposed complexes in D-F. Structures are shown from the highest (G) to the lowest similarity (I), using the distance between the two PAM-distal duplexes as a benchmark.

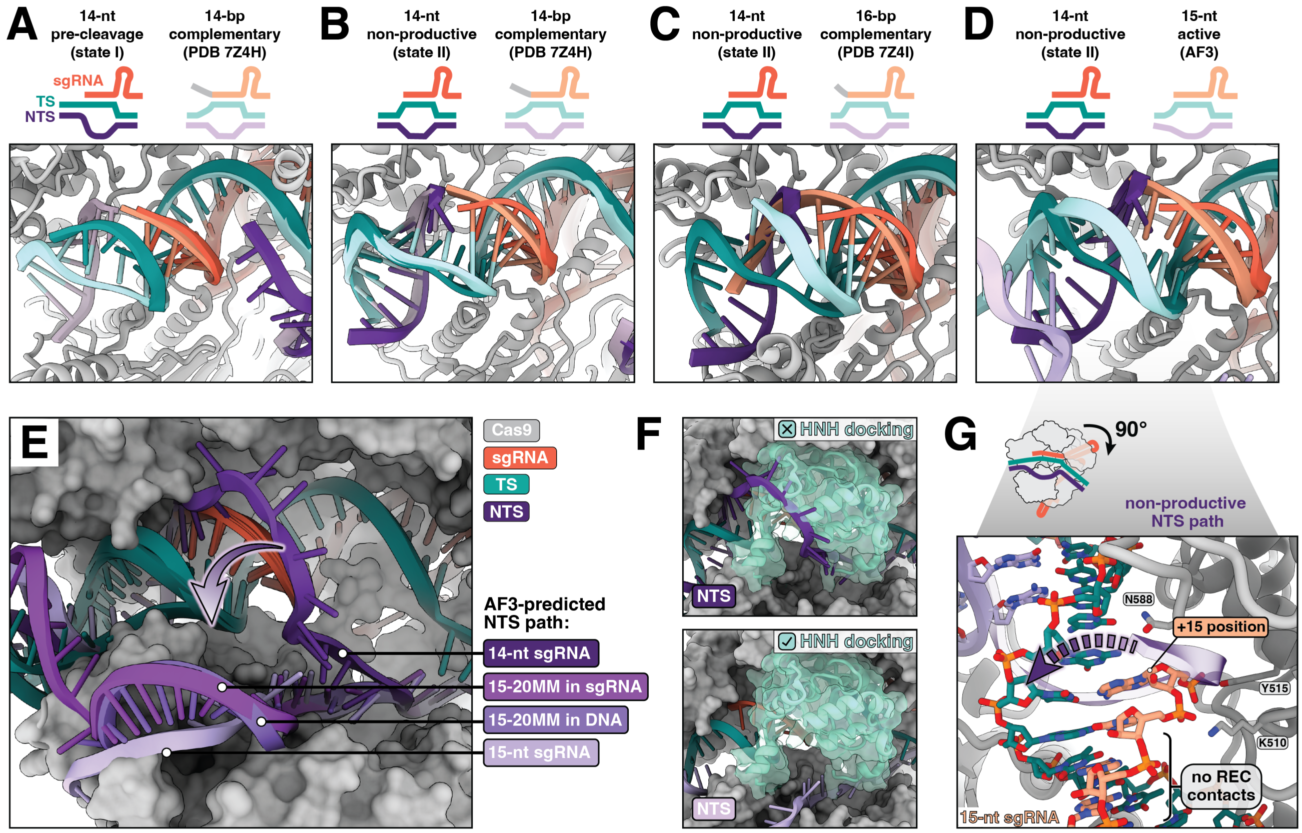

Supplementary Figure 2. Structural comparison of Cas9 bound to different R-loop lengths. (A) Overlay of the pre-cleavage structure with the structure of Cas9 bound to an R-loop containing 14-bp of complementarity. (B-D) Overlay of the non-productive structure with the structure of Cas9 bound to an R-loop containing (B) 14-bp of complementarity, (C) 16-bp of complementarity, and (D) an AlphaFold 3 (AF3) predicted structure containing a 15-nt truncated sgRNA. (E) Zoom-in view of the displaced NTS path in AF3 structures of Cas9 bound to a 14-nt sgRNA (same as in this study), an sgRNA containing 6 consecutive mismatches in positions +15-20 (same as in PDB 7Z4H), an sgRNA containing 6 mismatches in the DNA target in positions +15-20 (same as the bubbled substrate used in this study), and a 15-nt truncated sgRNA. (F) Overlay of HNH docked onto the TS from the active state structure PDB 7S4X. In the 14-nt sgRNA complex, the NTS is positioned to sterically occlude HNH docking (top panel). In the complexes with longer R-loops, the NTS path would allow HNH docking (bottom panel). (G) A close-up view of the non-productive state overlayed with the predicted 15-nt sgRNA structure. REC3 residues (K510, Y515, and N588) are within H-bonding distance from the sgRNA nucleotide in position +15. The NTS path (purple arrow) observed in our non-productive structure occupies this same space. The addition of another base pair, as in the 15-bp R-loop, would preclude the NTS from taking this path.

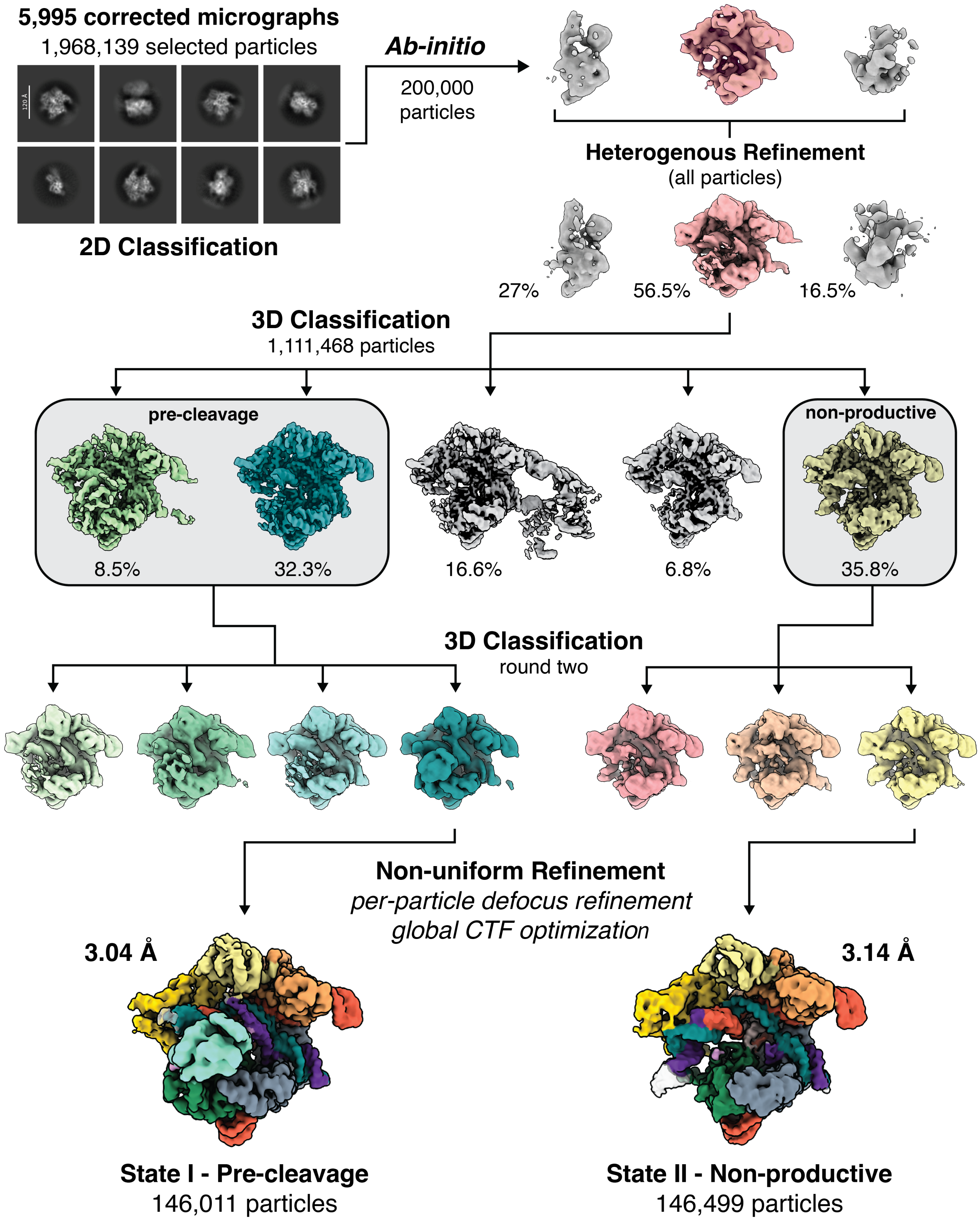

Supplementary Figure 3. Cryo-EM data processing workflow.

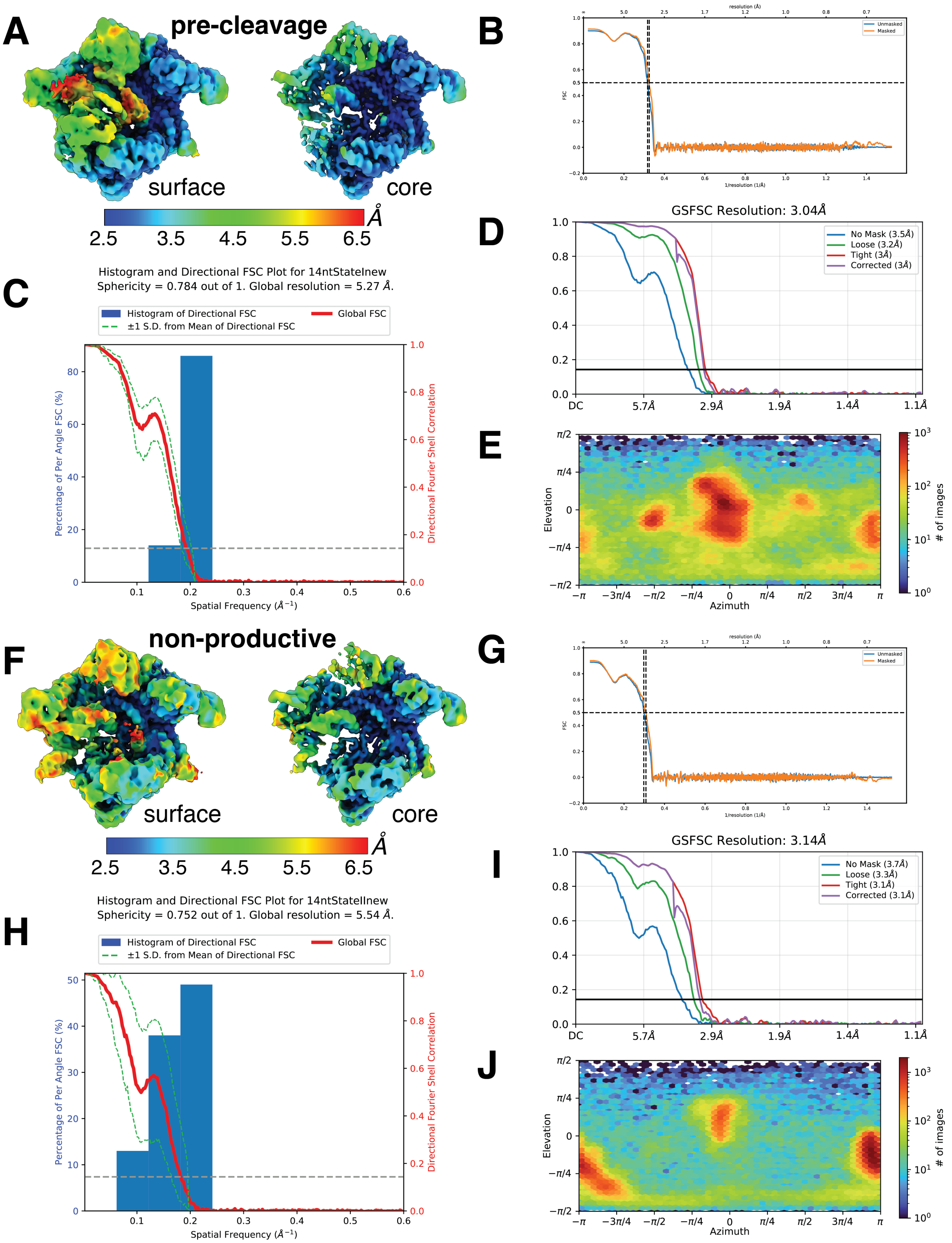

Supplementary Figure 4. Cryo-EM analysis. (A) Local resolution of pre-cleavage map shown at conservative (surface) and stringent (core) thresholds. (B) Pre-cleavage map-to-model FSC curves. (C) 3DFSC for pre-cleavage map. (D) FSC curves of pre-cleavage reconstruction. (E) Angular distribution plot for reconstruction of pre-cleavage state. (F) Local resolution of non-productive map shown at conservative (surface) and stringent (core) thresholds. (G) Non-productive state map-to-model FSC curves. (H) 3DFSC curve for non-productive map. (I) FSC for non-productive state. (J) Angular distribution plot for reconstruction of non-productive state.

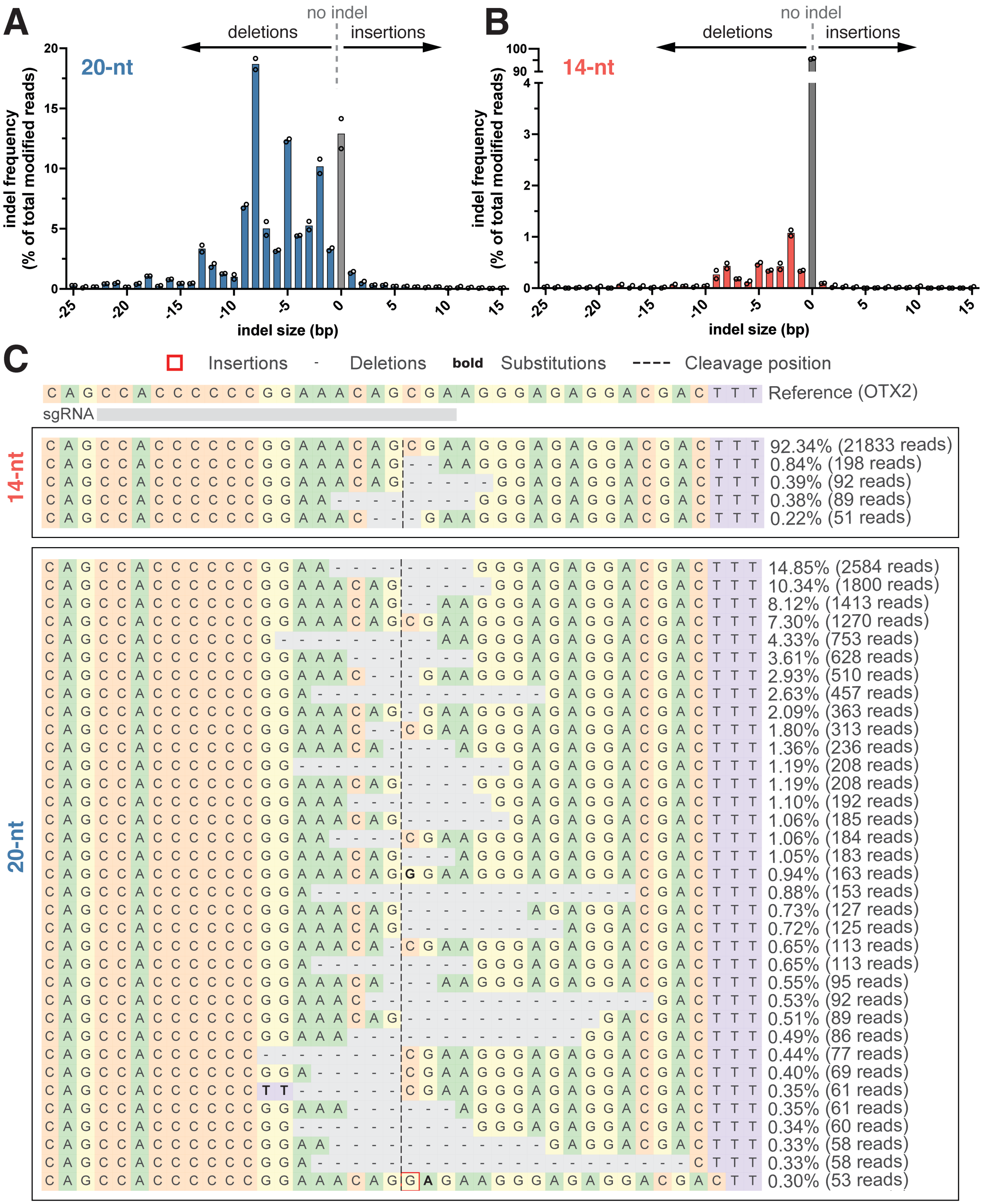

Supplementary Figure 5. Indel analysis at OTX2 locus. (A-B) Distribution of indel frequency from 20-nt edited (A) and 14-nt edited (B) samples. Data from two replicates are plotted as % of total indels and sorted by size of insertions (positive values) or deletions (negative values). (C) OTX2 genomic target site showing the reference sequence (top) and the modified sequences resulting from 14-nt or 20-nt-mediated editing.

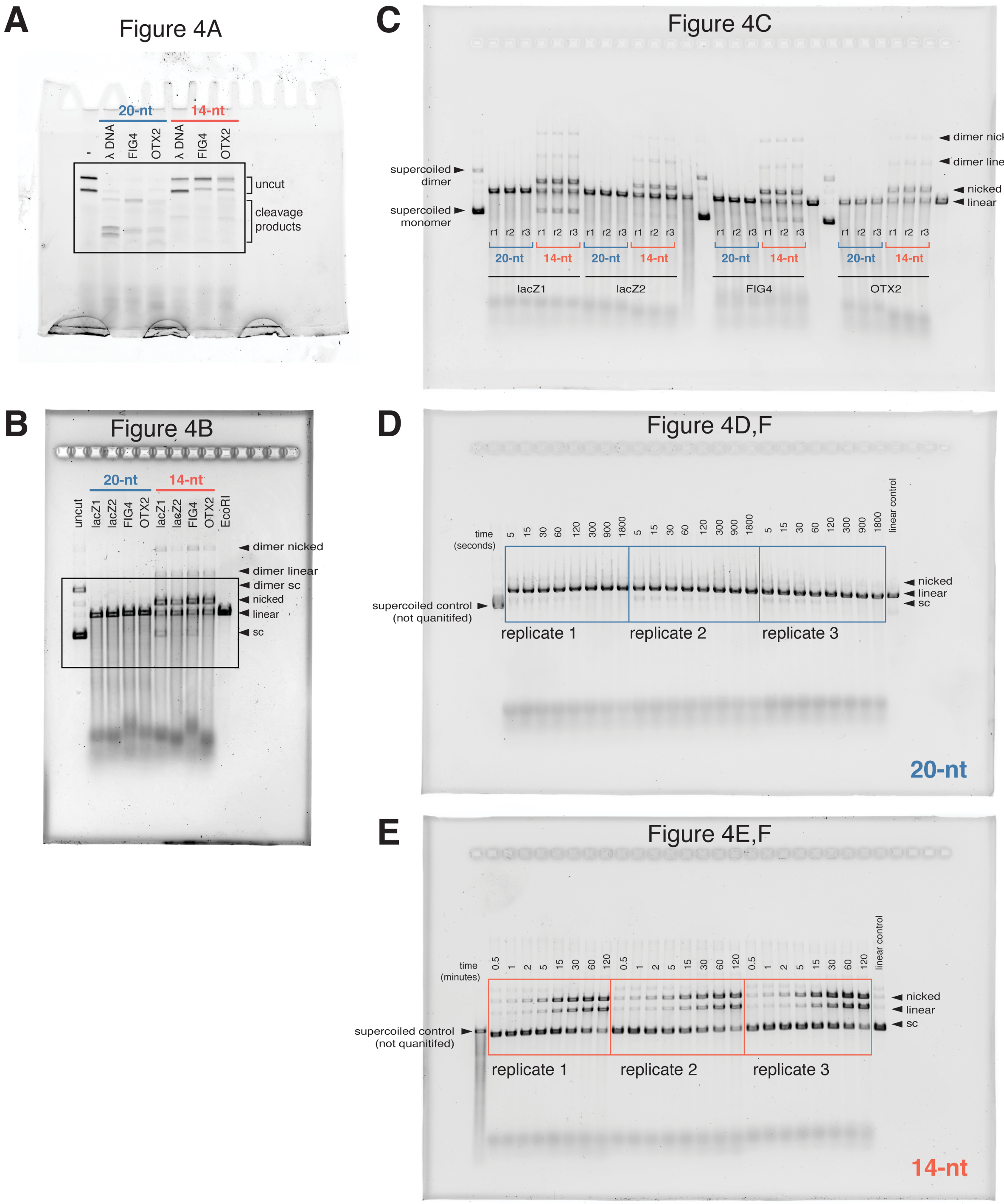

Supplementary Figure 6. Uncropped gel images. (A) Gel in Figure 4A showing cleavage of 55-bp linear DNA fragments using a 20-nt or 14-nt sgRNA after 18 hours of incubation. (B) Gel used in Figure 4B showing plasmid cleavage of four targets using 20-nt or 14-nt sgRNAs after 18 hours of incubation. (C) Gel containing replicates (n = 3) used for quantification of plasmid cleavage in Figure 4C after 18 hours of incubation. (D) Gel containing replicates (n = 3) used to determine plasmid cleavage rate with a 20-nt sgRNA and plotted in Figure 4D,F. Timepoints were taken at 5, 15, 30, 60, 120, 300, 900, and 1800 seconds. (E) Gel containing all replicates (n = 3) used to determine plasmid cleavage rate with a 14-nt sgRNA and plotted in Figure 4E,F. Timepoints were taken at 0.5, 1, 2, 5, 15, 30, 60, and 120 minutes.

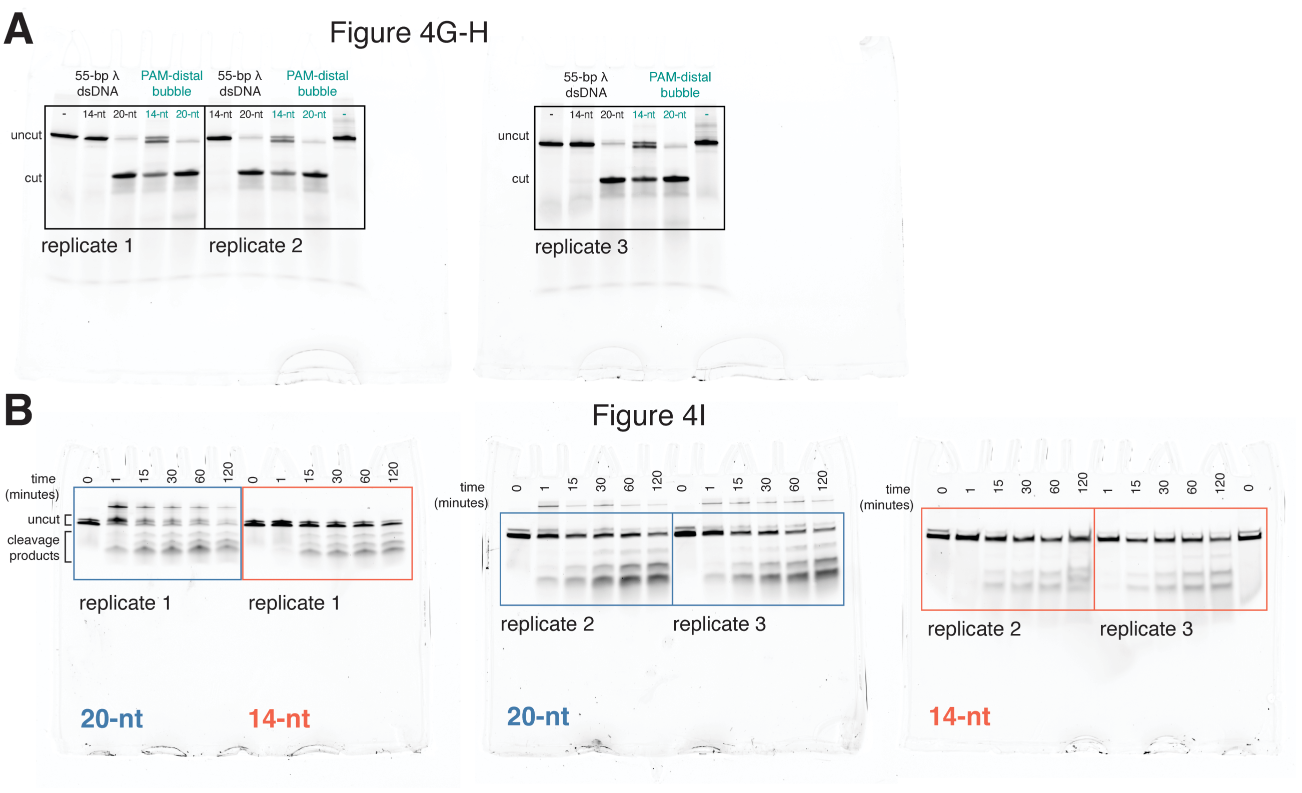

Supplementary Figure 7. Uncropped gel images (continued). (A) Gels of all replicates (n = 3) used to quantify Cas9 cleavage after 2 hours of incubation with a 55-bp dsDNA fragment and a 55-bp dsDNA fragment containing a PAM-distal bubble in positions +15-20, as shown in Figure 4G and Figure 4H. (B) Gels with all replicates (n = 3) showing Cas9 cleavage of a PAMmer substrate with a 20-nt or 14-nt sgRNA at 0, 1, 15, 30, 60, and 120 minutes. Product generated over time is plotted in Figure 4I. All quantification was performed in ImageJ and plotted in GraphPad Prism v10.3.1.

Name	Sequence (5'-3')	Source
40-bp λ TS	CTAATCGCCAGCGTCTCATCTTTATGCGCCTACGCACTGG	IDT
40-bp λ NTS	CCAGTGCGTAGGCGCATAAAGATGAGACGCTGGCGATTAG	IDT
Cy5-40-bp λ NTS	/Cy5/CCAGTGCGTAGGCGCATAAAGATGAGACGCTGGCGATT AG	IDT
55-bp λ TS	AGCTGACGTTTGTACTCCAGCGTCTCATCTTT ATGCGTCAGCAGAGATTTCTGCT	IDT
55-bp λ NTS	/56-FAM/AGCAGAAATCTCTGCTGACGCATAAAGATGAGACGC TGGAGTACAAACGTCAGCT	IDT
15-20MM λ NTS (bubbled)	/56-FAM/AGCAGAAATCTCTGCTCTGCGTTAAAGATGAGACGCT GGAGTACAAACGTCAGCT	IDT
PAMmer TS	/56-FAM/AGCTGACGTTTGTACTCCAGCGTCTCATCTTTATGCG TCAGCAGAGATTTCTGCT	IDT
PAMmer NTS	TGGAGTACAAACGTCAGCT	IDT
FIG4 TS	ATGAGCCAGAAAGGATCCTCCGGGTTGTTTCGAGCCGTCTCA GCTTTTGGAGTCG	IDT
FIG4 NTS	/56-FAM/CGACTCCAAAAGCTGAGACGGCTCGAAACAACCCGG AGGATCCTTTCTGGCTCAT	IDT
OTX2 TS	GTAAATGTCGTCCTCTCCCTTCGCTGTTTCCGGGGGGTGGCT GCAGGACAAGAAG	IDT
OTX2 NTS	/56-FAM/CTTCTTGTCCTGCAGCCACCCCCCGGAAACAGCGAA GGGAGAGGACGACATTTAC	IDT
sgRNA scaffold	(Spacer)-GUUUUAGAGCUAGAAAUAGCAAGUUAAAAUAAGGC UAGUCCGUUAUCAACUUGAAAAAGUGGCACCGAGUCGGUG CUUUU	Genscript

Supplementary Table 1. DNA substrates and synthetic sgRNA scaffold used in this study.

Target	20-nt spacer (5'-3')	14-nt spacer (5'-3')	Experiment
FIG4	GACGGCTCGAAACAACCCGG	TCGAAACAACCCGG	*in vivo* mESC editing
OTX2	CCACCCCCCGGAAACAGCGA	CCCGGAAACAGCGA	
ARNT	GTGAAATAGAACGGCGGCGA	TAGAACGGCGGCGA	
GAPDHS	GGTGGCTGTTCTTCAACCGT	TGTTCTTCAACCGT	
RAB7	GTCATCATCCTGGGGGACTC	ATCCTGGGGGACTC	
VPS54	GTCCTGCAATTCGTGTTGAG	CAATTCGTGTTGAG	
FBXW7	GCAAGGAGATGAAGTCTCGC	AGATGAAGTCTCGC	
ATP2B1	AGCTGCGATCCTCTTGTCGG	GATCCTCTTGTCGG	
VPS18	AGGCTAGTGATCCGCTCCGA	GTGATCCGCTCCGA	
BNIP3	CAAGAGTTCTCACTGTGAC	GTTCTCACTGTGAC	
ACTBL2	GGTTGGTCGTCCACGACACC	TCGTCCACGACACC	
NUTM2	ATCTTCACAGCGTCTTCACT	ACAGCGTCTTCACT	
λ-DNA	GGCGCATAAAGATGAGACGC	TAAAGATGAGACGC	cryo-EM
lacZ1	CAGCTGGCGTAATAGCGAAG	GCGTAATAGCGAAG	*in vitro* cleavage assays
lacZ2	CGAAAGGGGGATGTGCTGCA	GGGGATGTGCTGCA	
FIG4	GACGGCTCGAAACAACCCGG	TCGAAACAACCCGG	
OTX2	CCACCCCCCGGAAACAGCGA	CCCGGAAACAGCGA	

Supplementary Table 2. Spacer sequences used in this study.

Target	Fwd primer (5'-3')	Rev Primer (5'-3')
FIG4	acactgacgacatggttctacaGGGAGGCACACTTCTTACCT	tacggtagcagagacttggtctCTTAGTGGGATGGTGCCTCA
OTX2	acactgacgacatggttctacaACCGAGGCAAAAGTCCTGT	tacggtagcagagacttggtctGGTCTTGGCAAACAGAGCTT
ARNT	acactgacgacatggttctacaCACCTAGAATGGAGCCTCTTTC	tacggtagcagagacttggtctCTTGTCTGGTTTTCGAGCCA
GAPDHS	acactgacgacatggttctacaGTCTGGCTCTATCTGGGGTG	tacggtagcagagacttggtctCTCACCATCCCCACGTCTTA
RAB7	acactgacgacatggttctacaCTCCCGACAGCTTCAAGGAT	tacggtagcagagacttggtctCAGAGCCGAATGTGGAACTG
VPS54	acactgacgacatggttctacaACAGCCTGGGTAGTTTTCTTG	tacggtagcagagacttggtctAAAGGATTTGGATAGTCGAGAGT
FBXW7	acactgacgacatggttctacaTGCTTACAGGCTGTGGTTATG	tacggtagcagagacttggtctCGGACCAGAGAAGTTGCTG
ATP2B1	acactgacgacatggttctacaTCTGTTGGGGAAGAAGAAGGT	tacggtagcagagacttggtCTCCTGCTCAATTCGACTCTGC
VPS18	acactgacgacatggttctacaGCAGTGTATCCTTGCCCAG	tacggtagcagagacttggtctGGTATGTAAGTGCCCACCTG
BNIP3	acactgacgacatggttctacaGTTCCAGCCTCCGTCTCTAT	tacggtagcagagacttggtctCCTCTAGACTTGAGCCCCAG
ACTBL2	acactgacgacatggttctacaGTGCTGTGTTCCCCTCCAT	tacggtagcagagacttggtctAAGGTCAAGATGCCTCGCTT
NUTM2	acactgacgacatggttctacaCCAGTTTCCACTGACTACC	tacggtagcagagacttggtctCAAAATAACTGGGGTACTGGC

Supplementary Table 3. Primers used for amplicon-based NGS (Illumina adaptors in lowercase).

Data Collection	State I<br>'pre-cleavage'	State II<br>'non-productive'
Movies	5,995	5,995
Magnification	81,000	81,000
Voltage (kV)	300	300
Electron exposure (e^-/Å^2)	50	50
Defocus range (μM)	-1.0 to -2.5	-1.0 to -2.5
Pixel size (Å)	0.56	0.56
Symmetry imposed	C_1	C_1
Initial particle images	1,968,139	1,968,139
Final particle images	146,011	146,499
Map resolution (Å)	3.04	3.14
FSC threshold	0.143	0.143
Map resolution range (Å)	2.5-7	2.5-7
**Refinement**	**State I**	**State II**
Initial model (PDBID)	6O0X	7S4V
Model resolution (Å)	3.1	3.2
FSC threshold	0.5	0.5
Map sharpening *B* factor (Å^2)	-119.0	-116.5
Model Composition		
Non-hydrogen atoms	12,603	11,623
Protein residues	1,339	1,119
Nucleotides	139	144
Ligands	0	0
Mean *B* factors (Å^2)		
Protein	156.54	156.52
Nucleotides	156.77	161.69
R.m.s. deviations		
Bond lengths (Å)	0.003	0.004
Bond angles (°)	0.698	0.895
Validation		
MolProbity score	1.32	1.67
Clash score	3.00	6.88
Poor rotamers (%)	0.24	0.59
Ramachandran plot		
Favored (%)	96.47	95.85
Allowed (%)	3.53	4.15
Disallowed (%)	0	0

Supplementary Table 4. Cryo-EM data collection, processing, and structure refinement statistics
